# Quality of life in elderly with dizziness

**DOI:** 10.1590/S1808-86942010000600016

**Published:** 2015-10-19

**Authors:** Nancy Akemi Takano, Silvia Sper Cavalli, Maurício Malavasi Ganança, Heloísa Helena Caovilla, Mônica Alcantara de Oliveira Santos, Érica de Toledo Piza Peluso, Fernando Freitas Ganança

**Affiliations:** 1 Master's degree in Neuromotor Rehabilitation Science, Sao Paulo Bandeirante University. Physical therapist; 2 Master's degree in Neuromotor Rehabilitation Science, Sao Paulo Bandeirante University. Physical therapist; 3 Full professor of otorhinolaryngology, UNIFESP-EPM. Professor of the master's degree program on Rehabilitation of Body Balance and Social Inclusion, UNIBAN; 4 Associate professor, Otorhinolaryngology and Head & Neck Surgery Department, UNIFESP-EPM. Speech therapist; 5 Masters degree in Otorhinolaryngology, Medical School of the Sao Paulo Santa Casa; 6 Masters degree in Otorhinolaryngology, Medical School of the Sao Paulo Santa Casa; 7 Post-doctoral degree, UNIFESP-EPM. Psychologist. Post-doctoral degree, UNIFESP - EPM. Adjunct professor. Head of the Otology and Otoneurology Discipline, UNIFESP - EPM. Sao Paulo Federal University and Sao Paulo Bandeirante University (Universidade Federal de São Paulo e Universidade Bandeirante de São Paulo)

**Keywords:** vestibular diseases, aged, quality of life, dizziness, vertigo

## Abstract

**Abstract:**

Dizziness is frequent in elderly people.

**Aims:**

To evaluate the Quality of Life (QoL) in elderly subjects with dizziness, relate it with gender and age.

**Material and Method:**

A prospective study comprising 120 elderly patients with dizziness evaluated with Brazilian versions of the Whoqol-bref and the dizziness handicap inventory (DHI). The factor analysis (FA), the Mann Whitney and Kruskal Wallis tests, and the Spearman correlation were applied to study the results.

**Results:**

The most compromised domains were the DHI physical domain and the Whoqol-bref physical and environment domains. FA resulted in 3 factors in the DHI and 5 factors in the Whoqolbref. There was a moderate correlation (-0.596) in the total scores of both instruments. Males had a better QoL in the “environment perception and introspectivity” and “health perception” factors of the Whoqol-bref test. Females had a better QoL in the “functionality perception” factor of the Whoqol-bref test. There were no significant age differences.

**Conclusions:**

Elderly patients with dizziness have a worse QoL. Elderly females with dizziness have worse QoL scores in “environment perception and introspectivity” and “health perception” and better QoL in the “functionality perception” factor compared to elderly males.

## INTRODUCTION

Dizziness is a feeling of altered body balance. It occurs when the sensory information from the vestibular, visual and proprioceptive systems are in conflict.^1^ Dizziness is the most common symptom in the elderly;^2^ it originates in the vestibular system in 85% of cases.^3^

Dizziness affects the quality of life (QOL) of elderly patients in several manners; it may limit certain head and body movements, and jeopardize professional, domestic, social and/or leisure activities. Autonomy may be lost, resulting in dependence, fear, depression, anxiety, and withdrawal.^2^

Few studies on the QOL of patients with vestibular disease have been published.^4^ Assessing the QOL has become part of the work to establish the overall impact of diseases and treatments from the patient's perspective.

The most common definition of QOL is given by the World Health Organization (WHOQOL group) as: “individuals’ perceptions of their position in life in the context of the culture and value systems in which they live and in relation to their goals, expectations, standards and concerns.”^5^

Generic or specific questionnaires on the QOL of patients are applied to measure such subjectivity.

The WHOQOL-bref is a generic questionnaire that encompasses several dimensions of QOL; it helps describe the impact of any disease on the lives of patients, compare population, and assess the efficacy of therapies.^6^

The Dizziness Handicap Inventory (DHI) is a specific questionnaire focusing on the impact of dizziness on the QOL.^7^, ^8^

Applying specific (DHI) and generic (WHOQOL-bref) questionnaires on a given population of patients with vestibular disease may improve the assessment of loss in these patients because of these conditions, and may help establish correlations between both tools. No published studies in which the DHI and the WHOQOL were applied in the same sample population of elderly patients with vestibular disease were found.

Therefore, the purpose of this study was to assess the QOL of elderly patients with vestibular disease by applying the WHOQOL-bref and DHI questionnaires and to analyze the results with factor analysis, thereby correlating both tests according to gender and age.

## METHOD

A cross-sectional exploratory study was designed; it was approved by the institutional review board of the university institution in which it was undertaken (protocol no. 05/2006).

The sample comprised 120 consecutive elderly subjects (age > 65 years) seen at the otoneurology outpatient unit, presenting vestibular dysfunction and dizziness for at least two months. Patients using medication for treating diseases of the vestibular system and patients with psychiatric or neurological conditions were excluded from the sample.

All patients read and signed a free informed consent form. Subjects underwent a clinical evaluation consisting of the medical history, an otorhinolaryngological physical examination, audiometry, immittance testing, and vestibular testing (vectonystagmography), based on Ganança et al's criteria.^3^

The Brazilian versions of the DHI^2^ and the WHOQOL^9^ questionnaires were applied by trained interviewers to subjects with vestibular disease, on the same day as the medical assessment; there was no pre-established order, no words were explained and no synonyms were given.

The Brazilian DHI is a specific questionnaire consisting of 25 questions on three domains: physical (7 questions), emotional (9), and functional (9). Higher scores mean a more significant impact of dizziness on the QOL.

The WHOQOL-bref is a generic questionnaire consisting of 26 questions on the following domains: physical (7 questions), psychological (6), environment (8), social relations (3), and 2 general question on QOL. Answers are placed on a satisfaction scale; higher scores mean a better QOL.

Factor analysis was applied to the questionnaire results to reduce the number of variables. The Varimax method with the Kaiser normalization was applied using the SPSS (Statistical Package for the Social Sciences) version 12.0 software. The Bartlett and Kaiser-Meyer-Olkin (KMO) sphericity test was applied to test the adequacy of the factor analysis method for the data. The Cronbach alpha coefficient was used to test the internal reliability of data. Cronbach alpha coefficient values within 0.600 and 0.800 are considered adequate to assure data reliability and instrument quality in an exploratory study. Following the rotation method, the main variables composing each factor were selected depending on the factor loads, which were considered for analysis if over 0.300 (minimum cutoff value).^10^ Analysis of factor items at times requires discarding questionnaire assertions to improve the quality of the instrument. The assertion is discarded and the Cronbach alpha coefficient is again calculated to check its trend. The process is repeated until an adequate coefficient value is reached.^10^

The KMO and Bartlett's sphericity tests indicate the expected confidence level in data when treated with factor analysis.^10^

The Mann Whitney and Kruskal Wallis tests were applied to check for statistically significant differences respectively in gender and factors, and age and factors. The Spearman correlation was applied to measure the degree of relationship between factors and the total score.

The maximally acceptable significance level was 5% (a=0.05).

## RESULTS

### The sample

The age of patients ranged from 65 to 91 years (mean 73.9 years). There were 34 male and 86 female subjects. The majority of male subjects were aged 65 to 70 years, then 71 to 75 years. The majority of female subjects were aged 76 to 80 years.

Table 1 shows the means of answers in each domain of the Brazilian DHI, classified according to gender. Table 2 shows the same, but classified according to age.

**Table 1:** Mean scores of 120 elderly patients with vestibular disease in each domain of the Brazilian dizziness handicap inventory, according to gender.

	Domains of the Brazilian dizziness handicap inventory		
Gender	Emotional	Functional	Physical
Male	38,2	45,6	49,4
Female	38,6	44,8	53,2
Mean	38,5	45,0	52,1

**Table 2:** Mean scores of 120 elderly patients with vestibular disease in each domain of the Brazilian dizziness handicap inventory, according to age.

	Domains of the Brazilian dizziness handicap inventory		
Age	Emotional	Functional	Physical
65 a 70	42,3	46,8	55,6
71 a 75	35,3	43,5	48,7
76 a 80	36,2	41,0	50,6
≥ 81	42,9	57,6	55,8
Mean	38,5	45,0	52,1

The highest scores in all domains of the Brazilian DHI were found in the group aged 81 years or more.

Table 3 shows the means of the WHOQOL-bref domains, classified according to gender. Table 4 shows the same classified according to age. The lowest values were found in the physical domain of subjects aged 81 years or more.

**Table 3:** Mean scores of 120 elderly patients with vestibular disease in each domain of the WHOQOL-bref questionnaire, according to gender.

	WHOQOL domains				
Gender	General	Physical	Psychological	Environmental	Social
Male	54,0	53,7	63,2	48,3	63,5
Female	51,6	51,0	55,7	52,3	62,0
Mean	52,3	51,8	57,9	51,1	62,4

**Table 4:** Mean scores of 120 elderly patients with vestibular disease in each domain of the WHOQOL-bref questionnaire, according to age.

	WHOQOL domains				
	General	Physical	Psychological	Environmental	Social
65 a 70	50,0	53,4	59,0	50,0	59,8
71 a 75	56,6	55,1	57,6	51,6	58,0
76 a 80	49,0	48,8	55,2	51,0	66,0
≥ 81	58,0	45,8	63,6	54,3	73,1
Mean	52,3	51,8	57,9	51,1	62,4

### Factor analysis of the Brazilian DHI results

The value of the KMO test was 0.84; the significance was zero in Bartlett's sphericity test. Thus, there was no probability of error in rejecting the null hypothesis.

Factor analysis with the Varimax orthogonal rotation of the Brazilian DHI yielded three factors, as follows: (1) compromised mental structure, (2) physical limitation e (3) loss of function. Taken together, these factors answered for 44% of the variance.

After the relevant items were extracted and selected, we applied the Cronbach alpha coefficient to evaluate the internal consistency of each factor and to assess a new alpha factor for any factor that was eliminated from the instrument. All factors were internally consistent.

### Factor analysis of the WHOQOL-bref results

The value of the KMO test was 0.81; the significance was zero in Bartlett's sphericity test. Thus, there was no probability of error in rejecting the null hypothesis.

There were five factors in the factor analysis of the WHOQOL-bref questionnaire, as follows: (1) perception of the environment and introspectiveness; (2) global and structural perception; (3) perception of functionality; (4) perception of health; and (5) perception of social relationships. These factors taken together answered for 52% of the variance. The Cronbach alpha coefficient values were internally consistent.

### Spearman's correlation analysis - Brazilian DHI and WHOQOL-bref

The Spearman's correlation coefficient indicated a moderate correlation (-0.596) between the total scores of both instruments. The negative value indicates score inversions in the answers of one instrument relative to the other.

### Mann Whitney test analysis - gender X Brazilian DHI and WHOQOL-bref factors

WHOQOL-bref factors 1, 3, and 4 were statistically significant. There were no statistically significant differences in the remaining variables.

Male subjects had better QOL scores in WHOQOL-bref factors 1 (71 males and 49 females) and 4 (70 males and 50 females). Female subjects scored better in factor 3 (46 males 60 females).

### Kruskal Wallis test analysis - age group X Brazilian DHI and WHOQOL-bref factors

There were no significant differences among factors in both instruments for all age groups.

## DISCUSSION

The notion of QOL is absolutely personal; each patient provides a different perception in each symptomatological dysfunction. Guyatt et al.^11^ have corroborated this idea by explaining that two patients with the same disease often perceive the condition differently. Perceptions about QOL change depending on the circumstances.^12^

A few authors have applied generic and specific questionnaires jointly to assess the QOL in a given population.^13^, ^14^, ^15^ However, only Gámiz and Lopez-Escamez^16^ applied some of these instruments to elderly patients with vestibular disease.

Of 120 subjects who answered the questionnaires, 86 were female and 34 were male. This gender distribution is similar to that in a study by Gámiz and Lopez-Escamez;^16^ these authors point to the fact that dizziness is more common in female elderly subjects with vestibular disease.

There were more subjects aged 65 to 70 years, as in the studies of Ramos et al.^17^ who found that 58% of the elderly population in Southeast Brazil is aged less than 70 years.

The study population comprised patients who sought medical care in the public healthcare system; most of them had little formal education (15 illiterate elderly subjects and 80 with basic education). Perceptions about QOL would probably have been different in a private institution and a population with higher purchasing power and education. In a study entitled “Rehabilitation care from the elderly patient´s perspective,” Kramer^18^ found that financial incentives are directly proportion with the perception of patients about their health and lives. This author also found that wealthier and more educated elderly subjects tend to use their time interacting with the social milieu and becoming involved in pleasurable activities.

Female subjects had higher means in all domains of the Brazilian DHI compared to males in the present study. These results concur with those of Robertson and Ireland^19^ in that vestibular disease affects more the lives of women than of men. This was observed in all domain of the WHOQOL-bref in our study except in the environmental domain, which may have occurred because the items of this subscale pertain to concerns about the basic structural means for living. These differences should be taken into account in patients with vestibular diseases for a more adequate therapeutic approach according to gender.

Patients aged over 81 years in our study scored higher in the functional, emotional, and physical aspects of the DHI (higher means) and on the physical aspect of the WHOQOL-bref (lower means); this is probably because the sensory systems involved with body balance and sensory-motor integration deteriorate with age.^16^ The physical domains of the Brazilian DHI and the WHOQOL-bref were the most affected domains, as confirmed in the literature.^13^, ^16^, ^19^, ^20^

The functional domain of the Brazilian DHI was also affected, as also found by Whitney et al.;^21^ these authors stated that dizziness may affect activities of daily living by altering the ability to perform these tasks.

The emotional and psychological domains were also affected, as also found in studies by Enloe and Shields^14^ and Asmundson et al.;^22^ these authors reported several psychoaffective manifestation related to vestibular disease.

The domain “social relationships” was the least affected WHOQOL-bref domain. Several authors, however, have pointed at the loss of this domain in patients with dizziness. Robertson and Ireland^19^ stated that this domain is directly related with dizziness, and that patients with vestibular disease reduce their social interactions because of phobias and fear of falls.

The domain “environment” was significantly affected in elderly patients with vestibular disease. The questions in this domain include situations or activities pertaining to the losses or inabilities generated by dizziness. In the WHOQOL-bref, we found that elderly patients scored the aspect “environment” as one of the most relevant.^23^

Our study also showed that patients reported worse QOL because of dizziness mainly in the “environmental” and “physical” domains of the WHOQOL-bref and in the physical domain of the Brazilian DHI.

The item 26 of the WHOQOL-bref specifically analyzes negative feelings such as: poor mood, despair, anxiety, and depression. A poor mood generally occurs because of physical limitations caused by vertigo.^24^

Three factors were found in the Brazilian DHI, and five factors were found in the WHOQOL-bref, respectively with 44% and 52% of the total variance in each instrument. Factors 1, 2, and 3 were “compromised mental structure”, “physical limitation” and “loss of function”. Factors 1, 2, 3, 4, and 5 were “perception of the environment and introspectiveness”, “global and structural perception”, “perception of functionality”, “perception of health” and “perception of social relationships”.

Questions 8 (Does performing more ambitious activities like sports, dancing, household chores such as sweeping or putting dishes away increase your problem?), 14 (Because of your problem, is it difficult for you to do strenuous housework or yardwork?) and 25 (Does bending over increase your problem?) in the Brazilian DHI were the items that best represented the constructs. Its relevance for elderly patients is that vestibular disease restricts activities of daily living and some bodily movements - especially movements of the head - because of dizziness.^16^ Cavalli's^25^ conclusion (using factor analysis) was that representativeness was adequate, particularly for difficult activities such as sports, dancing and doing housework. Jacobson and Newman^14^ confronted these results with the original validation of the instrument and indicated questions 3 (Because of your problem, do you restrict your travel for business or recreation?), 17 (Does walking down a sidewalk increase your problem?) and 18 (Because of your problem, is it difficult for you to concentrate?) as the most representative. Knowing these more relevant items may help guide the diagnosis.

Perez et al.^26^ also applied the Varimax factor analysis to assess the results of the DHI in 337 patients aged from 14 to 83 years with peripheral vestibular dysfunction. Variance in the DHI was 48.32%; the factors were “vestibular handicap, vestibular disability and visuo-vestibular disability”. The items that best represented the instrument were questions 13 (Does turning over in bed increase your problem?), 14 (Because of your problem, is it difficult for you to do strenuous housework or yardwork?) and 21 (Because of your problem, do you feel handicapped?). Comparing these findings with our work showed that items tended to cluster around factors 1 and 2.

Asmundson et al.^22^ presented possible attempts to groups the DHI items under different factors. These authors studied 95 patients of an otoneurology clinic to investigate the factorial structure of the DHI. Factor analysis of the instrument was also done, but with oblimin rotation. Two factors were extracted, “functional limitation”, which comprised 23 items, and “postural difficulty”, which comprised 2 items; the variance was 47.9%. A second attempt by these authors resulted in three factors being extracted, namely “activities of daily living handicap”, comprising 14 items, “phobia”, comprising 9 items, and “postural difficulties”, comprising 2 items; the variance was 53.8%.

Enloe and Shields^14^ applied the DHI and the Medical Outcomes Study 36-Item Short Form (SF-36), respectively a specific and a generic instrument, to a sample of 95 subjects with vestibular dysfunction. Both instruments did not correlate highly, but the authors explained that the questionnaires focus on different evaluation purposes, and that when used jointly, they are able to supplement and enrichen the medical assessment of vestibular disease. The emotional domain scored highest (62.45) among the DHI domains, which differed from our study where the physical domain scored highest (52.1).

The Varimax factor analysis method with orthogonal rotation was applied in developing the WHOQOL-100; six domains were identified, as follows: “physical”, “psychological”, “independence level”, “social relationships”, “environment”, “spirituality”, and general questions on QOL that related to all factors. The domain variance was 58%.

In our study, the WHOQOL-bref questions that best represented elderly patients with vestibular disease were: 13 (How available to you is the information that you need in your day-to-day life?), 18 (How satisfied are you with your capacity for work?) and 22 (How satisfied are you with the support you get from your friends?). The current pace of automation, information and communication affect the life experience; thus, studying the abovementioned difficulties in the elderly population supports appropriate care and concerns about the QOL of these patients.

The pilot test with the WHOQOL-bref contained 17 questions that were significantly more important for female subjects; for male subjects, only the item “sex life” was relevant; possibly, female subjects felt intimidated when answering this last item. Some items were more important in the elderly population, such as: “environment”, “social support”, “transportation”, “feeling of physical safety”, “care with health”, and “activities of daily living”.^23^

Most studies using the WHOQOL-bref questionnaire consisted of subjects from the general population - patients from several medical areas and age groups - whereas our study included only elderly subjects with vestibular disease. The factor analysis results for the WHOQOL-bref questionnaire vary depending on the study population.

Male subjects had a better QOL than female subjects in the WHOQOL-bref factors “perception of the environment and introspectiveness” and “perception of health”, while female subjects had a better QOL in the factor “perception of functionality”. The factor results above, as applied to male subjects, may be so because men tend not to admit vulnerability in several life aspects, thereby yielding the results we encountered. Female elderly subjects perceive better QOL issues pertaining to the factor “perception of functionality”, probably because of the habitual and dedicated behavior of housework.

There were no significant differences between gender and the DHI factors, probably because of the specific nature of this questionnaire. Generic instruments deal with topics involving several aspects that measure QOL in general, which bring male-female differences to the fore. This fact supports the idea that applying both tools adds to the quality of the clinical history.

No significant differences between age and the factors were found, showing that dizziness affected QOL regardless of the age of patients with vestibular disease.

In our study, Spearman's correlation for the WHOQOL-bref and Brazilian DHI factors revealed a low relationship between domains, possibly because of the types of questionnaires (generic or specific) and the difference between the variables of each instrument. The total correlation of these instruments was moderate (0.59), indicating a reasonable relation between the two for assessing the QOL of elderly patients with vestibular disease. These findings concur with those of Fielder et al.^13^ in a study of 42 vertigo patients using the SF-36 and the DHI; these authors found moderate correlations between domains (> 0.53). These results suggest that one questionnaire does not exclude the other, although both may be applied to elderly subjects. The fact that the WHOQOL-bref questionnaire is generic for QOL assessments and that the Brazilian DHI is specific for vestibular disease means that associating both may be an additional approach for the care of this population, which has decreased QOL as one of the main consequences of disease.

There is no doubt that the topic QOL is polemic and cannot be discussed fully in such a simplified manner. These debates will certainly continue to take up a significant time in the main conferences and studies on this topic. Our results demonstrate the losses in several aspects of the QOL of elderly patients with vestibular disease and underline the importance of applying specific and generic instruments in this population.

## CONCLUSIONS

Applying the Brazilian versions of the DHI and WHOQOL-bref questionnaires on 120 elderly subjects with vestibular dysfunction and chronic dizziness led to the following conclusions:

1. The QOL of these patients is impaired.
2. The factors that we identified were: “compromised mental structure”, “physical limitation” and “loss of function” in the Brazilian DHI, and “perception of the environment and introspectiveness”, “global and structural perception”, “perception of functionality”, “perception of health” and “perception of social relationships” in the WHOQOL-bref.
3. Loss of QOL as established in the total score of the Brazilian DHI is associated with loss of QOL as detected in the total score of the WHOQOL-bref questionnaire.
4. Female elderly subjects with vestibular disease had a worse QOL in the factors “perception of the environment and introspectiveness” and “perception of health” and had better QOL scores in the factor “perception of functionality” compared with male subjects.
5. The QOL of elderly patients with chronic dizziness does not depend on age.
